# Prognostic Value of Right Ventricular Performance and Left Atrial Mechanical Efficiency in Paroxysmal Atrial Fibrillation

**DOI:** 10.3390/jcdd13060269

**Published:** 2026-06-15

**Authors:** Aristi Boulmpou, Efstathios Pagourelias, Georgios Zormpas, Dimitrios Ntelios, Vassilios Vassilikos, Christodoulos Papadopoulos

**Affiliations:** 1 Third Department of Cardiology, Aristotle University of Thessaloniki, Ippokratio General Hospital, 546 42 Thessaloniki, Greece; 2 Second Department of Cardiology, Aristotle University of Thessaloniki, Ippokratio General Hospital, 546 42 Thessaloniki, Greece

**Keywords:** atrial fibrillation, paroxysmal, prognosis, echocardiography, right ventricle, left atrial strain

## Abstract

**Background**: Predicting atrial fibrillation (AF) recurrence remains a major clinical challenge, as conventional echocardiographic parameters often fail to capture the complex electro-mechanical substrate of the arrhythmia. The prognostic significance of right ventricular (RV) function and atrial mechanical–structural coupling in paroxysmal AF (PAF) remains underexplored. **Methods**: We prospectively enrolled patients with PAF in sinus rhythm undergoing comprehensive echocardiography. A wide range of conventional left-sided, right-sided, and novel coupling indices was assessed. Univariable analysis was performed to screen for potential AF recurrence predictors. Based on the initial findings, receiver operating characteristic (ROC) analysis was used to determine the optimal cutoff for RV fractional area change (RV FAC). Finally, multivariable logistic regression identified independent predictors of AF recurrence over a 12-month follow-up. **Results**: A total of 73 patients were included, of whom 31 (42.5%) experienced AF recurrence during 12-month follow-up. Conventional left atrial (LA) indices, including LA volume index (LAVI) and reservoir strain, showed no significant association with recurrence. In univariable analysis, RV FAC, LA contraction strain, and the novel LA contraction strain/LAVI ratio were all significant predictors. ROC analysis identified an RV FAC cutoff of 42.5%, with lower values associated with significantly higher recurrence rates. In multivariable analysis, lower RV systolic performance determined by RV FAC ≤ 42.5% emerged as a primary independent predictor of recurrence (*p* = 0.003), while the LA contraction strain/LAVI ratio demonstrated a strong trend towards significance (*p* = 0.076). **Conclusions**: In this exploratory study of patients with PAF, atrial mechanical–structural mismatch emerged as a primary marker of the arrhythmic substrate. Additionally, an exploratory signal suggested that a subclinical reduction in RV performance may also correlate with recurrence, though this warrants further investigation in larger cohorts.

## 1. Introduction

Atrial fibrillation (AF) remains the most prevalent sustained arrhythmia and is associated with substantial morbidity, impaired quality of life, and increased healthcare costs [1]. Despite advances in rhythm control strategies, including optimized medical therapy and catheter ablation, predicting AF recurrence continues to represent a major clinical challenge [2]. Accurate identification of patients at high risk for recurrence is essential for treatment selection, follow-up intensity, and optimization of outcomes [3].

Echocardiography has long been central to AF risk stratification, with particular emphasis on left atrial (LA) size, transmitral filling indices, and, more recently, LA strain [4,5]. Numerous studies have highlighted the role of LA reservoir or conduit strain as a surrogate of atrial remodeling and compliance [6]. However, these indices are often influenced by loading conditions, rhythm variability, and image quality, limiting their reproducibility and generalizability [7]. Moreover, conventional parameters frequently fail to discriminate which patients with paroxysmal AF (PAF) will progress to recurrence, suggesting that current approaches incompletely capture the mechanical substrate that predisposes to arrhythmia [8,9].

Beyond the left atrium, there is increasing recognition that AF is not solely a left-sided disease. Right ventricular (RV) function and biventricular mechanical substrate are emerging contributors to atrial remodeling and arrhythmogenesis [10,11]. Yet, the prognostic significance of RV systolic performance in PAF remains underexplored, and right heart parameters are rarely incorporated into recurrence prediction models.

Furthermore, recent research has shifted focus toward coupling parameters, namely indices that characterize the efficiency of the interaction between cardiovascular compartments (such as the atrio-ventricular or ventriculo-arterial interfaces) rather than assessing isolated chambers [12,13,14]. While coupling has been well-studied in heart failure, its application to the mechanical–structural interplay of the LA remains limited [15]. Specifically, this coupling represents the relationship between atrial contractile force and chamber enlargement. It is based on the concept that as the atrium dilates, it must generate greater contractile strain to maintain its booster-pump function. It is hypothesized that integrating atrial function with atrial size could provide a more physiological marker of how well the atrium compensates for early remodeling, yet this concept is highly unexplored in patients with PAF.

Our group recently reported exploratory findings from the PLACEBO trial, which suggested that while higher galectin-3 and SDRR are independent predictors of AF recurrence, right ventricular fractional area change (RV FAC) demonstrated only a marginal independent association [16]. This raised the critical pathophysiological question whether RV dysfunction could be a standalone predictor or reflects a broader, integrated mechanical substrate. Consequently, the present analysis was performed to investigate whether more refined right-sided parameters or integrated markers of atrial mechanical–structural coupling, which account for the interplay between contractile strain and chamber volume, could provide further prognostic support than isolated conventional indices.

To address this gap, the present study aimed to investigate the prognostic value of conventional echocardiographic parameters, right heart functional indices, and coupling markers in a homogeneous cohort of patients with PAF. Particular focus was placed on evaluating whether integrating RV function and refined atrial mechanics could provide a more physiological approach to predicting AF recurrence during a 12-month follow-up.

## 2. Materials and Methods

### 2.1. Study Design

This study is an echocardiographic sub-analysis from the PLACEBO trial (NCT05246423), a prospective, observational, single-center cohort study examining predictors of AF recurrence using echocardiography, laboratory biomarkers, cardiopulmonary exercise testing, and autonomic indices. The full study protocol has been published elsewhere [16]. The main study was conducted at the Third Department of Cardiology, Ippokratio General Hospital, Aristotle University of Thessaloniki, Greece, and received approval from the Institutional Review Board and the Aristotle University of Thessaloniki Ethics Committee. All patients provided written informed consent in accordance with the Declaration of Helsinki.

### 2.2. Study Population

Study participants were adults aged 18 years or older with a documented history of PAF who were in sinus rhythm during the baseline evaluation. PAF was defined according to the 2020 and 2024 ESC Guidelines as AF episodes that terminate spontaneously or with intervention within 7 days, typically within 48 h [2,17]. To ensure stable rhythm and minimize the influence of acute atrial mechanical stunning on echocardiographic measurements, patients were enrolled only if at least 2 weeks had elapsed since their most recent documented AF episode. AF diagnosis and episode timing were verified through hospital or emergency department electrocardiographic (ECG) recordings, ambulatory 24 h ECG Holter monitoring, and review of medical records detailing both onset and termination of arrhythmia.

Alcohol consumption was defined as current regular intake, specified as at least one standard alcoholic unit per week during the year preceding enrollment, as reported during the baseline clinical interview.

Exclusion criteria comprised: i. persistent or permanent AF, ii. structural cardiomyopathy, iii. congenital heart disease, iv. left ventricular ejection fraction (LVEF) < 50%, v. moderate or severe valvular disease, vi. recent acute coronary syndrome, vii. recent major surgery, viii. end-stage renal disease, ix. autoimmune or malignant disease, x. overt or uncontrolled thyroid or hypertensive disorders, xi. prior AF ablation, xii. pregnancy, or xiii. inadequate echocardiographic image quality. Patients unable to provide written informed consent or to comply with follow-up procedures were also excluded. These criteria ensured the creation of a homogeneous cohort free of major structural or systemic confounders.

### 2.3. Follow-Up

All participants were followed for 12 months after enrollment. AF recurrence was assessed during scheduled clinical visits through standard 12-lead ECGs, repeat 24 h ECG Holter monitoring, and systematic review of hospital or outpatient medical records. When patients reported symptoms suggestive of arrhythmia, additional ECG recordings were performed. AF recurrence was defined as any documented AF episode lasting at least 30 s, consistent with guideline-based definitions and the primary outcome definition of the PLACEBO trial [2,16,17].

### 2.4. Echocardiographic Assessment

A comprehensive transthoracic echocardiogram was performed in all participants in sinus rhythm using a GE Vivid E95 ultrasound system (GE Healthcare, Chicago, IL, USA), following standardized acquisition protocols. All examinations were performed by a single experienced cardiologist, and measurements were analyzed in accordance with current EACVI/ASE recommendations [18,19,20].

LA maximum, minimum, and pre-A volumes were obtained using the biplane disk summation method from apical four- and two-chamber views. The LA volume index (LAVI) was calculated by indexing the maximum LA volume to body surface area. LA strain analysis, including reservoir, conduit, and contraction components, was performed using two-dimensional speckle-tracking echocardiography from the apical four-chamber view at frame rates between 50 and 80 frames per second. To characterize atrial mechanical–structural coupling, we calculated a novel index: the LA contraction strain/LAVI ratio. This parameter was derived to assess atrial contractile efficiency normalized to the degree of structural remodeling.

Left ventricular (LV) volumes and systolic function were assessed using the Simpson’s biplane method. LV diastolic function was evaluated using transmitral Doppler flow (E and A velocities), tissue Doppler imaging of the mitral annulus, and the E/e′ ratio.

Right-heart assessment included measurement of RV FAC from traced end-diastolic and end-systolic areas in the apical four-chamber view, along with tricuspid annular plane systolic excursion (TAPSE) obtained via M-mode. Pulmonary artery systolic pressure (PASP) was estimated from the tricuspid regurgitant jet velocity combined with an estimate of right atrial (RA) pressure. RA volume was assessed using the area-length method. In addition to these conventional indices, several coupling parameters were evaluated specifically for this analysis, including the RV FAC/RA volume ratio, the TAPSE/PASP ratio as a measure of RV–pulmonary arterial coupling, and the left atrial coupling index (LACI), defined as the ratio of minimum LA volume to left ventricular end-diastolic volume. All echocardiographic parameters were measured across three cardiac cycles and averaged.

To ensure the objectivity of the data, all echocardiographic and strain analyses were performed at baseline, prior to the completion of the 12-month clinical follow-up. Consequently, the investigator remained blinded to the eventual AF recurrence status of the patients during the quantification of all echocardiographic indices. To assess measurement stability, intra-observer variability was evaluated in a random subset of 10 patients, demonstrating high reproducibility.

**Figure 1:**
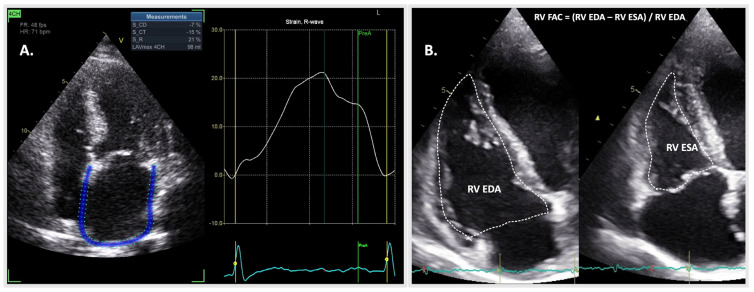
Methodology for right ventricular and left atrial assessment. (**A**). Measurement of left atrial strain using speckle-tracking echocardiography from the apical four-chamber view with the corresponding strain curve. (**B**). Assessment of right ventricular fractional area change (RV FAC), derived from RV end-diastolic area (EDA) and end-systolic area (ESA) tracings in apical four-chamber view. RV FAC was calculated as (RV EDA − RV ESA)/RV EDA. Both parameters were significant predictors of arrhythmia recurrence in the present cohort.

Figure 1 illustrates how LA strain and RV FAC were measured using speckle-tracking echocardiography and RV area tracing, respectively.

### 2.5. Statistical Analysis

Continuous variables were assessed for normality using the Shapiro–Wilk test and are presented as mean ± standard deviation (SD) or median with interquartile range (IQR), depending on distribution. Categorical variables are presented as counts and percentages. Differences between patients with and without AF recurrence were evaluated using the independent samples *t*-test or Mann–Whitney U test for continuous variables, and the χ^2^ test or Fisher’s exact test for categorical variables. Correlations between non-normally distributed continuous variables were evaluated using Spearman’s rank correlation coefficient.

To identify predictors of AF recurrence, univariable logistic regression was first performed for all echocardiographic variables and coupling indices. Variables with a *p*-value below 0.10 were considered marginally significant and were subsequently entered into a multivariable logistic regression model to determine independent predictors. Odds ratios (OR) with corresponding 95% confidence intervals were calculated. Multicollinearity was assessed using variance inflation factors. Receiver operating characteristic (ROC) curve analysis was performed to determine the optimal cutoff value for RV FAC in predicting AF recurrence. Based on the highest Youden index, patients were dichotomized into two groups (RV FAC > 42.5% vs. ≤42.5%). Additionally, time-to-recurrence was explored using Kaplan–Meier survival curves and compared via the Log-rank test. To further assess the temporal risk associated with RV function, a univariable Cox proportional hazards model was constructed to calculate the hazard ratio (HR) for the dichotomized RV FAC groups.

A two-sided *p*-value < 0.05 was considered statistically significant. Statistical analyses were performed using SPSS (version 23, IBM Corp., Armonk, NY, USA).

## 3. Results

A total of 73 patients with PAF were included in the analysis, of whom 31 (42.5%) experienced AF recurrence during the 12-month follow-up period. Baseline demographic and clinical characteristics were comparable between patients with and without AF recurrence (Table 1). The duration of AF history did not differ between groups. Obstructive sleep apnea (OSA) was more prevalent among patients with recurrence; however, this finding did not significantly affect subsequent analyses. Overall, the cohort was homogeneous, reflecting the restricted inclusion criteria and the absence of major structural heart disease.

Baseline echocardiographic indices were similar across both groups, with no significant differences found in LV dimensions or ejection fraction (Table 2). LAVI was within normal limits for the total cohort and did not differ significantly between those with and without recurrence [28(8) vs. 31(6) mL/m^2^, *p* = 0.324]. However, patients who experienced AF recurrence had significantly higher absolute LA contraction strain (12 ± 4% vs. 9 ± 4%, *p* = 0.034). Furthermore, RV systolic performance was significantly lower in the recurrence group, as evidenced by a lower RV FAC [40.5(9.3)% vs. 44.6(6.1)%, *p* = 0.008].

Conventional echocardiographic parameters, including LAVI, LA emptying fraction (LAEF), E/e′ ratio, and LA reservoir or conduit strain, did not differ significantly between groups and were not associated with AF recurrence in univariable analyses. Similarly, atrioventricular and ventriculo-atrial coupling markers, such as LACI, the RV FAC/RA volume ratio, and the TAPSE/PASP ratio, did not demonstrate predictive value. Furthermore, Spearman’s rank correlation analysis revealed no significant correlation between absolute LA contraction strain and the E/e′ ratio (rho = 0.064, *p* = 0.599), suggesting that the observed alterations in atrial contractile function were independent of estimated LV filling pressures.

Three variables emerged as significant in univariable logistic regression: lower RV FAC, reduced LA contraction strain, and a higher LA contraction strain/LAVI ratio (Table 3). RV FAC showed a negative association with AF recurrence (OR 0.918; 95% CI 0.854–0.987; *p* = 0.020), indicating that impaired RV systolic performance was linked to a greater likelihood of arrhythmic relapse. LA contraction strain was also inversely associated with recurrence (OR 0.886; 95% CI 0.790–0.994; *p* = 0.039), while the LA contraction strain/LAVI ratio exhibited a positive association (OR 12.238; 95% CI 1.294–115.775; *p* = 0.029), suggesting that disproportionate preservation of LA contractile function relative to atrial size may reflect underlying mechanical–structural mismatch.

**Figure 2:**
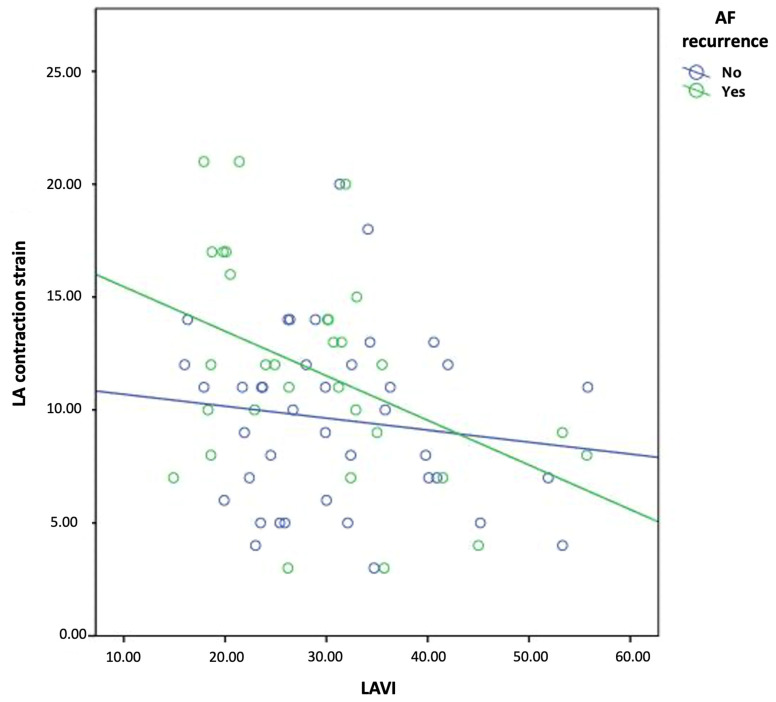
Scatter plot demonstrating LA mechanical–structural mismatch. The relationship between LAVI (X-axis) and absolute LA contraction strain (Y-axis) is plotted for patients with (green) and without (blue) AF recurrence. The distinct regression slopes illustrate that for any given atrial volume, patients in the recurrence group exhibit an altered, augmented contractile deformation. This geometric separation visually captures the compensatory atrial wall stress and mechanical–structural mismatch that predisposes patients to AF recurrence, independent of isolated chamber dilation.

To further visualize the dynamic relationship between left atrial deformation and structural chamber size, a bivariate scatter plot analysis was performed, mapping absolute LA contraction strain against LAVI (Figure 2). The analysis revealed distinct geometric trajectories between the two cohorts. Patients who experienced AF recurrence clustered along a markedly steeper regression slope compared to those without recurrence. This demonstrates that for any given left atrial volume, the recurrence cohort exhibited an augmented, disproportionate contractile response.

In multivariable analysis, RV FAC remained the only independent predictor of AF recurrence (OR 0.921; 95% CI 0.853–0.994; *p* = 0.034). Notably, the LA contraction strain/LAVI ratio demonstrated a strong trend towards significance (OR 14.769; 95% CI 0.806–84.792; *p* = 0.076). LA contraction strain alone did not retain significance after adjustment for the other variables. Despite its higher prevalence in the recurrence group (48.4% vs. 23.8%, *p* < 0.05), OSA was not included in the multivariable model in order to maintain a stable events-per-variable ratio and to avoid model overfitting, prioritizing the evaluation of novel echocardiographic markers.

To further evaluate the discriminative ability of RV FAC, ROC curve analysis was performed. RV FAC demonstrated significant predictive value for recurrence (AUC = 0.686; 95% CI 0.557–0.815; *p* = 0.008). The Youden Index identified an optimal cutoff value of 42.5%. At this threshold, RV FAC predicted recurrence with a sensitivity of 73.3% and a specificity of 71.8%. When patients were stratified by this cutoff, those with reduced RV FAC (≤42.5%) exhibited a markedly higher rate of recurrence compared to those with higher RV FAC (66.7% versus 22.5%, respectively, *p* < 0.001). Patients with an RV FAC ≤ 42.5% had nearly a 7-fold increase in the odds of AF recurrence (OR = 6.89; 95% CI 2.44–19.42) (Supplementary Figure S1).

Finally, the timing of AF recurrence was explored using survival analysis. Kaplan–Meier curves (Supplementary Figure S2) illustrated that patients with RV FAC ≤ 42.5% showed a descriptive trend toward earlier recurrence compared to those with higher values, although the difference did not reach statistical significance in the Log-rank test (*p* = 0.348) or the univariable Cox proportional HR model (HR 1.38; 95% CI 0.63–3.02; *p* = 0.416).

## 4. Discussion

In this prospective cohort of patients with PAF, we found that conventional LA indices and atrioventricular coupling parameters did not predict arrhythmic recurrence during a 12-month follow-up period. Instead, two findings emerged as key markers of vulnerability: an index expressing the proportional relationship between LA contraction strain and LA size, and secondary reductions in RV systolic performance, as reflected by RV FAC. Together, these observations highlight the potential importance of right-sided cardiac function while exploring atrial mechanical–structural interactions in the early substrate of PAF.

The lack of association between traditional parameters such as LAVI, E/e′ ratio, or LA reservoir strain and AF recurrence aligns with reports suggesting that these indices may better reflect advanced atrial remodeling rather than the subtle structural–functional alterations present in the early stages of the disease [21,22]. A series of prior studies addressing LA strain have focused on heterogeneous AF populations or patients already scheduled for catheter ablation, where atrial fibrosis and remodeling are more pronounced [23,24]. In contrast, our highly selected cohort consisted of patients with generally preserved cardiac structure and no major comorbidities, which may explain why standard markers performed poorly. This reinforces the concept that in early AF, reliance on LA reservoir strain alone may overlook subtle mechanical alterations relevant to recurrence risk.

Instead, the relationship between recurrence and LA contraction strain provides an additional layer of insight into atrial mechanics. Although LA contraction strain did not remain independently predictive after adjustment, the concept of mechanical–structural mismatch, rooted in the Frank–Starling mechanism, proved highly informative. In the early stages of AF, the atrium often undergoes subtle eccentric remodeling. As visualized in our scatter plot analysis (Figure 2), patients with recurrence clustered along a significantly steeper regression slope. This mathematically confirms that for any given LA volume, the recurrent cohort generated an augmented, disproportionate contractile response. This suggests a compensatory state where the atrial myocardium acts as an enhanced booster pump to maintain stroke volume despite early chamber enlargement. However, this state of chronic mechanical compensation leads to increased atrial wall stress and metabolic demand, precursors to electrical remodeling and trigger activity. Thus, this geometric mismatch identifies an atrium functioning at the limit of its mechanical reserve, creating a vulnerable substrate even before overt fibrosis is detectable by conventional strain.

Furthermore, the clinical profile of the recurrence cohort, characterized by a higher prevalence of OSAS, female sex, and isolated LA myopathy, strongly resembles a subclinical HFpEF-like phenotype. While our cohort was not formally evaluated for clinical HFpEF via invasive hemodynamics or validated dyspnea scores, the augmented LA contractile strain observed prior to macroscopic dilation may represent an early atrial response to this systemic profile. Identifying these patients through advanced strain imaging may help explain the presence of a more hostile substrate that drives AF recurrence. Additionally, while OSA was more prevalent in the recurrence group, reflecting its known biological role in increasing right-sided pressures and atrial stretch, the primary focus of our analysis remained on identifying novel echocardiographic markers within the statistical constraints of our event rate.

Secondarily, exploratory analyses identified subclinical reductions in RV FAC as a potential marker of AF recurrence (Supplementary Material). It is important to emphasize that our derived cutoff lies within the clinically accepted normal range. Although AF is traditionally viewed as a predominantly left-sided arrhythmia, accumulating evidence indicates that RV afterload, RA dynamics, and pulmonary vascular interactions contribute to atrial arrhythmogenesis [25]. Studies from community cohorts and pulmonary hypertension populations have demonstrated that elevated RV pressure load or impaired RV function is associated with incident AF and its recurrence [26,27]. However, the fact that RV FAC predicted recurrence despite its moderate AUC (0.686) warrants careful interpretation. In a cohort characterized by minimal structural remodeling, early reductions in RV functional reserve may act as a sensitive surrogate marker for unmeasured left-sided diastolic stiffness and subsequent pulmonary vascular transmission, rather than acting as the primary driver of the arrhythmia.

Our findings collectively suggest that early AF recurrence is not driven by the parameters traditionally emphasized in AF management, such as LA size or reservoir strain, but rather by more subtle abnormalities in right-sided cardiac performance and atrial mechanical coupling. These observations are in line with recent interest in characterizing AF phenotypes beyond simple structural markers [28,29]. They also complement the principal aim of the PLACEBO study, which seeks to define multifactorial predictors of AF recurrence through integrative phenotyping approaches combining echocardiography, CPET, and biomarkers [16,30].

From a clinical perspective, our results suggest that the LA contraction strain/LAVI ratio may offer a more individualized measure of atrial vulnerability than LA strain alone, particularly in patients with PAF and preserved remodeling profiles. These findings demonstrate that it may not only be the LA that underlies the vulnerability to AF recurrence, but rather a more complex biventricular mechanical substrate where RV performance plays a complementary role. RV FAC is readily obtainable from standard echocardiographic views and does not require advanced imaging techniques. Similarly, these parameters could potentially guide follow-up intensity, rhythm control strategies, or the timing of advanced interventions; however, further validation is needed before clinical adoption.

This study should be interpreted in the context of several limitations. It represents a relatively small, single-center cohort, and although the population was intentionally homogeneous, the sample size and the number of recurrence events (n = 31) restricted the number of variables that could be included in the multivariable model. Strict adherence to the events-per-variable principle was maintained to ensure model stability; consequently, although OSA is a clinically relevant factor in AF pathophysiology, it could not be included in the multivariable analysis without risking statistical overfitting. Additionally, OSA was identified through clinical history rather than polysomnography, which may have led to an underestimation of its true prevalence. Second, the follow-up duration of 12 months may not fully capture long-term recurrence patterns, and the absence of continuous rhythm monitoring introduces the possibility of underdetecting asymptomatic AF episodes, although our standardized protocol of serial ECGs and 24 h Holter monitoring remains consistent with current guideline recommendations for rhythm follow-up [2]. Finally, while RV FAC was identified as a significant independent predictor, its moderate area under the curve (AUC = 0.686) reflects the complex, multifactorial nature of AF recurrence. This suggests that while RV performance is a significant independent predictor, no single echocardiographic index can provide perfect discrimination in a low-remodeling cohort, suggesting the need for future integrated models combining imaging, biomarkers, and clinical data. In parallel, given the exploratory nature of the study, a large number of echocardiographic variables were tested without formal correction for multiple comparisons; consequently, the isolated finding regarding RV FAC must be interpreted with caution, as there is a risk of a type I statistical error. Furthermore, as an exploratory study, it may have been underpowered to detect smaller effect sizes for parameters that did not reach the significance threshold, such as the LA contraction strain/LAVI ratio (*p* = 0.076). Therefore, our findings should be considered hypothesis-generating and require validation in larger, multicenter cohorts to confirm their generalizability across more diverse AF phenotypes. The prospective design of our study, with strict inclusion criteria, standardized echocardiographic acquisition, and uniform imaging conditions, strengthens the reliability of the identified predictors.

In conclusion, evidence of LA mechanical–structural mismatch, characterized by augmented contractile strain prior to macroscopic chamber dilation, appears to be a more relevant marker of early AF recurrence than conventional left-sided parameters. Furthermore, secondary subclinical alterations in RV systolic performance may act as early indicators of broader cardiopulmonary interplay. These findings highlight underrecognized aspects of AF pathophysiology. Larger multicenter studies are of paramount importance to validate these mechanical indices and determine their role in the personalized management of AF.

## Figures and Tables

**Table 1:** Baseline demographic characteristics and comorbidities of patients with and without atrial fibrillation recurrence. Abbreviations: AF, atrial fibrillation; BMI, body mass index.

Variable	No AF Recurrence (n = 42)	AF Recurrence (n = 31)	*p*-Value
Age (years)	58.69 ± 13.12	60.77 ± 9.36	0.454
BMI	28.24 ± 4.59	27.39 ± 4.07	0.376
Female sex	42.9%	54.8%	0.311
AF duration (months)	7.5 (2.8–58.3)	37 (8.0–60.0)	0.325
AF episode last month	38.1%	38.7%	1.000
Total cardioversions			
*0*	83.3%	80.6%	0.950
*1*	14.3%	16.1%	
*>2*	2.4%	3.2%	
Hypertension	57.1%	51.6%	0.639
Diabetes	7.1%	9.7%	0.697
Dyslipidemia	52.4%	48.4%	0.736
Heart failure	0.0%	3.2%	0.425
Coronary artery disease	4.8%	3.2%	1.000
Thyroid disease	26.2%	19.4%	0.582
Obstructive sleep apnea	2.4%	19.4%	0.037
Smoking	33.3%	29.0%	0.696
Alcohol	47.6%	54.8%	0.638
Exercise (any)	64.3%	74.2%	0.449
Beta-blocker use	66.7%	58.1%	0.452
Antiarrhythmic use	42.9%	51.6%	0.459
*Class I*	28.6%	41.9%	0.319
*Class III*	14.3%	9.7%	0.724
Beta-blocker + antiarrhythmic use	38.1%	41.9%	0.740

**Table 2:** Baseline echocardiographic characteristics of the study population. Data are presented as mean ± standard deviation or median (interquartile range) as appropriate. All LA strain values are reported as absolute values. Abbreviations: LVEDD, left ventricular end-diastolic diameter; LVEDV, left ventricular end-diastolic volume; LVESV, left ventricular end-systolic volume; LVEF, left ventricular ejection fraction; LAVI, left atrial volume index; RVEDV, right ventricular end-diastolic volume; RVESV, right ventricular end-systolic volume; RV FAC, right ventricular fractional area change; TAPSE, tricuspid annular plane systolic excursion.

Variable	No AF Recurrence (n = 42)	AF Recurrence (n = 31)	*p*-Value
LVEDD (mm)	47 ± 5	47 ± 5	0.574
LVEDV (mL)	110 ± 28	107 ± 32	0.610
LVESV (mL)	43 ± 14	41 ± 13	0.635
LVEF (%)	61.7 (7.0)	60.8 (8.7)	0.995
LAVI (mL/m^2^)	31 (6)	28 (8)	0.324
LA reservoir strain (%)	24 ± 7	26 ± 8	0.280
LA conduit strain (%)	14 ± 5	14 ± 5	0.833
LA contraction strain (%)	9 ± 4	12 ± 4	0.034
E (m/s)	0.81 (0.3)	0.78 (0.3)	0.719
A (m/s)	0.65 ± 0.18	0.67 ± 0.19	0.685
E/A	1.3 (0.8)	1.3 (0.5)	0.594
E/e′	8.7 (2.6)	7.9 (4.4)	0.405
RVEDV (mL)	36 ± 11	35 ± 11	0.703
RVESV (mL)	15 ± 6	15 ± 6	0.733
RV FAC (%)	44.6 (6.1)	40.5 (9.3)	0.008
PASP (mmHg)	24 ± 10	24 ± 9	0.988
TAPSE (mm)	26 ± 5	25 ± 4	0.401

**Table 3:** Univariable and multivariable predictors of atrial fibrillation recurrence. Abbreviations: OR, odds ratio; CI, confidence interval; LA, left atrial; LAVI, left atrial volume index; LACI, left atrial coupling index; LAEF, left atrial ejection fraction; RV FAC, right ventricular fractional area change; RA, right atrial; TAPSE, tricuspid annular plane systolic excursion; PASP, pulmonary artery systolic pressure.

Variable	Model Type	Exp(B) (OR)	95% CI	*p*-Value
LA reservoir strain (%)	Univariable	1.036	0.973–1.103	0.273
LA conduit strain (%)	Univariable	1.010	0.920–1.110	0.830
LA contraction strain (%)	Univariable	0.886	0.790–0.994	0.039
LAVI (mL/m^2^)	Univariable	0.978	0.931–1.027	0.370
LA contraction strain/LAVI (%/mL/m^2^)	Univariable	12.238	1.294–115.775	0.029
LACI (LA volume minimum/LVEDV)	Univariable	0.672	0.018–25.112	0.829
LAEF (%)	Univariable	1.024	0.974–1.077	0.352
E/e′ ratio	Univariable	0.950	0.834–1.083	0.446
RV FAC (%)	Univariable	0.918	0.854–0.987	0.020
RV FAC/RA volume (%/mL)	Univariable	1.023	0.264–3.967	0.974
TAPSE/PASP ratio (mm/mmHg)	Univariable	0.104	0.000–68.782	0.494
RV FAC (%)	Multivariable	0.921	0.853–0.994	0.034
LA contraction strain (%)	Multivariable	1.039	0.830–1.300	0.740
LA contraction strain/LAVI (%/mL/m^2^)	Multivariable	14.769	0.806–84.792	0.076

## Data Availability

The data presented in the study are available on request from the corresponding author. The data are not publicly available due to privacy restrictions of the Greek National Health System.

## Supplementary Materials

The following supporting information can be downloaded at: https://www.mdpi.com/article/10.3390/jcdd13060269/s1.

## Abbreviations

The following abbreviations are used in this manuscript:

AF	atrial fibrillation
LA	left atrial
PAF	paroxysmal atrial fibrillation
RV	right ventricular
ECG	electrocardiographic
LVEF	left ventricular ejection fraction
LAVI	left atrial volume index
LV	left ventricular
RV	FAC right ventricular fractional area change
TAPSE	tricuspid annular plane systolic excursion
PASP	pulmonary artery systolic pressure
RA	right atrial
LACI	left atrial coupling index
SD	standard deviation
IQR	interquartile range
OR	odds ratio
ROC	receiver operating characteristic
OSA	obstructive sleep apnea
LAEF	left atrial emptying fraction

## References

[1] Sukaina M., Waheed M., Rehman S., Hasibuzzaman M.A., Meghani R. (2025). Demographic trends in mortality with older population due to atrial fibrillation and flutter from 1999–2020. World J. Cardiol..

[2] Van Gelder I.C., Rienstra M., Bunting K.V., Casado-Arroyo R., Caso V., Crijns H.J.G.M., De Potter T.J.R., Dwight J., Guasti L., Hanke T. (2024). 2024 ESC Guidelines for the management of atrial fibrillation developed in collaboration with the European Association for Cardio-Thoracic Surgery (EACTS). Eur. Heart J..

[3] Desai N.R., Giugliano R.P. (2012). Can we predict outcomes in atrial fibrillation?. Clin. Cardiol..

[4] Asinger R.W. (2000). Role of transthoracic echocardiography in atrial fibrillation. Echocardiography.

[5] Cameli M., Mandoli G.E., Loiacono F., Sparla S., Iardino E., Mondillo S. (2016). Left atrial strain: A useful index in atrial fibrillation. Int. J. Cardiol..

[6] Olsen F.J., Diederichsen S.Z., Jørgensen P.G., Jensen M.T., Dahl A., Landler N.E., Graff C., Brandes A., Krieger D., Haugan K. (2024). Left Atrial Strain Predicts Subclinical Atrial Fibrillation Detected by Long-term Continuous Monitoring in Elderly High-Risk Individuals. Circ. Cardiovasc. Imaging.

[7] Silva M.R., Sampaio F., Braga J., Ribeiro J., Fontes-Carvalho R. (2023). Left atrial strain evaluation to assess left ventricle diastolic dysfunction and heart failure with preserved ejection fraction: A guide to clinical practice: Left atrial strain and diastolic function. Int. J. Cardiovasc. Imaging.

[8] Li G., Wang X., Han J.J., Guo X. (2022). Development and validation of a novel risk model for predicting atrial fibrillation recurrence risk among paroxysmal atrial fibrillation patients after the first catheter ablation. Front. Cardiovasc. Med..

[9] Saglietto A., Gaita F., Blomstrom-Lundqvist C., Arbelo E., Dagres N., Brugada J., Maggioni A.P., Tavazzi L., Kautzner J., De Ferrari G.M. (2022). AFA-Recur: An ESC EORP AFA-LT registry machine-learning web calculator predicting atrial fibrillation recurrence after ablation. EP Eur..

[10] Vijan A., Daha I.C., Delcea C., Dan G.A. (2023). The complex interplay between right ventricular dysfunction and atrial fibrillation—A narrative review. Rom. J. Intern. Med..

[11] Li S., Bi X., An Q., Li Y., Li C., Shen D. (2025). Right ventricular dysfunction improves prediction of atrial fibrillation in hypertrophic cardiomyopathy: A cardiac magnetic resonance study. Front. Cardiovasc. Med..

[12] Wu J., Zhao Y., Gu X., Chen B., Wu R., Andong A., Shi R., Wu C., Dai Y., Zhao L. (2026). Atrioventricular Coupling Index: A Novel Approach to Risk-Stratification for Major Adverse Cardiovascular Events in Patients With ST-Elevation Myocardial Infarction. J. Magn. Reson. Imaging.

[13] Li A., Zhang M., Ning B. (2024). Predictive value of the left atrioventricular coupling index for recurrence after radiofrequency ablation of paroxysmal atrial fibrillation. J. Cardiothorac. Surg..

[14] Gaczoł M., Olszanecka A., Bednarek A., Kusiak A., Kiełbasa G., Moskal P., Jastrzębski M., Sondej T., Stolarz-Skrzypek K., Rajzer M. (2024). Selected echocardiographic and blood pressure parameters, including ventricular-arterial coupling, in predicting recurrence of atrial fibrillation after pulmonary vein isolation: Preliminary study. Kardiol. Pol..

[15] Terlizzi V.D., Barone R., Nunno N.D., Alcidi G., Brunetti N.D., Iacoviello M. (2024). The Atrioventricular Coupling in Heart Failure: Pathophysiological and Therapeutic Aspects. Rev. Cardiovasc. Med..

[16] Boulmpou A., Moysiadis T., Zormpas G., Teperikidis E., Vassilikos V., Giannakoulas G., Papadopoulos C. (2025). Exploring the Feasibility of Integrating Cardiopulmonary Exercise Testing, Echocardiography, and Biomarkers for Predicting Atrial Fibrillation Recurrence: Rationale, Design and Protocol for a Prospective Cohort Study (The PLACEBO Trial). J. Clin. Med..

[17] Hindricks G., Potpara T., Dagres N., Arbelo E., Bax J.J., Blomström-Lundqvist C., Boriani G., Castella M., Dan G.-A., Dilaveris P.E. (2021). 2020 ESC Guidelines for the diagnosis and management of atrial fibrillation developed in collaboration with the European Association for Cardio-Thoracic Surgery (EACTS): The Task Force for the diagnosis and management of atrial fibrillation of the European Society of Cardiology (ESC) Developed with the special contribution of the European Heart Rhythm Association (EHRA) of the ESC. Eur. Heart J..

[18] Galderisi M., Cosyns B., Edvardsen T., Cardim N., Delgado V., Di Salvo G., Donal E., Sade L.E., Ernande L., Garbi M. (2017). Standardization of adult transthoracic echocardiography reporting in agreement with recent chamber quantification, diastolic function, and heart valve disease recommendations: An expert consensus document of the European Association of Cardiovascular Imaging. Eur. Heart J. Cardiovasc. Imaging.

[19] Thomas J.D., Edvardsen T., Abraham T., Appadurai V., Badano L., Banchs J., Cho G.-Y., Cosyns B., Delgado V., Donal E. (2025). Clinical Applications of Strain Echocardiography: A Clinical Consensus Statement From the American Society of Echocardiography Developed in Collaboration With the European Association of Cardiovascular Imaging of the European Society of Cardiology. J. Am. Soc. Echocardiogr..

[20] Badano L.P., Kolias T.J., Muraru D., Abraham T.P., Aurigemma G., Edvardsen T., D’Hooge J., Donal E., Fraser A.G., Marwick T. (2018). Standardization of left atrial, right ventricular, and right atrial deformation imaging using two-dimensional speckle tracking echocardiography: A consensus document of the EACVI/ASE/Industry Task Force to standardize deformation imaging. Eur. Heart J. Cardiovasc. Imaging.

[21] Floria M., Radu S., Gosav E.M., Cozma D., Mitu O., Ouatu A., Tanase D.M., Scripcariu V., Serban L.I. (2020). Left Atrial Structural Remodelling in Non-Valvular Atrial Fibrillation: What Have We Learnt from CMR?. Diagnostics.

[22] Vitarelli A. (2024). Left Atrial Volumes and Strain: Integrating Approach in Predicting Atrial Fibrillation and Recurrence after Ablation. Cardiology.

[23] Kim J., Park S.-J., Jeong D.S., Chung S., Jeon K., Bak M., Kim D., Kim E.K., Chang S.-A., Lee S.-C. (2023). Left atrial strain predicts fibrosis of left atrial appendage in patients with atrial fibrillation undergoing totally thoracoscopic ablation. Front. Cardiovasc. Med..

[24] Kuppahally S.S., Akoum N., Burgon N.S., Badger T.J., Kholmovski E.G., Vijayakumar S., Rao S.N., Blauer J., Fish E.N., DiBella E.V.R. (2010). Left atrial strain and strain rate in patients with paroxysmal and persistent atrial fibrillation: Relationship to left atrial structural remodeling detected by delayed-enhancement MRI. Circ. Cardiovasc. Imaging.

[25] Ruscio E., Gabrielli F.A., Pinnacchio G., Spera F.R., Giordano F., Scacciavillani R., Narducci M.L., Bencardino G., Perna F., Crea F. (2025). Right atrial strain in atrial fibrillation: The hidden side of the moon. Front. Cardiovasc. Med..

[26] Parikh R.R., Norby F.L., Wang W., Thenappan T., Prins K.W., Van’t Hof J.R., Lutsey P.L., Solomon S.D., Shah A.M., Chen L.Y. (2022). Association of Right Ventricular Afterload with Atrial Fibrillation Risk in Older Adults: The Atherosclerosis Risk in Communities Study. Chest.

[27] Le Quilliec E., LeBlanc C.-A., Neuilly O., Xiao J., Younes R., Altuntas Y., Xiong F., Naud P., Villeneuve L., Sirois M.G. (2024). Atrial cardiomyocytes contribute to the inflammatory status associated with atrial fibrillation in right heart disease. Europace.

[28] Woodman R.J., Ng H.S., Mangoni A.A. (2025). Clinical phenotypes of atrial fibrillation: A review of machine learning applications in personalized treatment. JRSM Cardiovasc. Dis..

[29] Zhang J., Wang L., Li Y., Li Q., Liu X., Weng S., Yin Y., Liang Z., Zhang T., Wang Y. (2025). Atrial fibrillation phenotypes identified through cluster analysis in the CABANA study. Int. J. Cardiol..

[30] Boulmpou A., Moysiadis T., Zormpas G., Teperikidis E., Tsioni K., Toumpourleka M., Zidrou M., Giannakoulas G., Vassilikos V., Papadopoulos C. (2025). Integrated Diagnostics for Atrial Fibrillation Recurrence: Exploratory Results from the PLACEBO Trial. Diagnostics.

