# Supplementary figures and images for: Prognostic Value of Right Ventricular Performance and Left Atrial Mechanical Efficiency in Paroxysmal Atrial Fibrillation
Source: J Cardiovasc Dev Dis. 2026 Jun 15;13(6):269. doi: 10.3390/jcdd13060269 (PMC13301432; doi:10.3390/jcdd13060269)

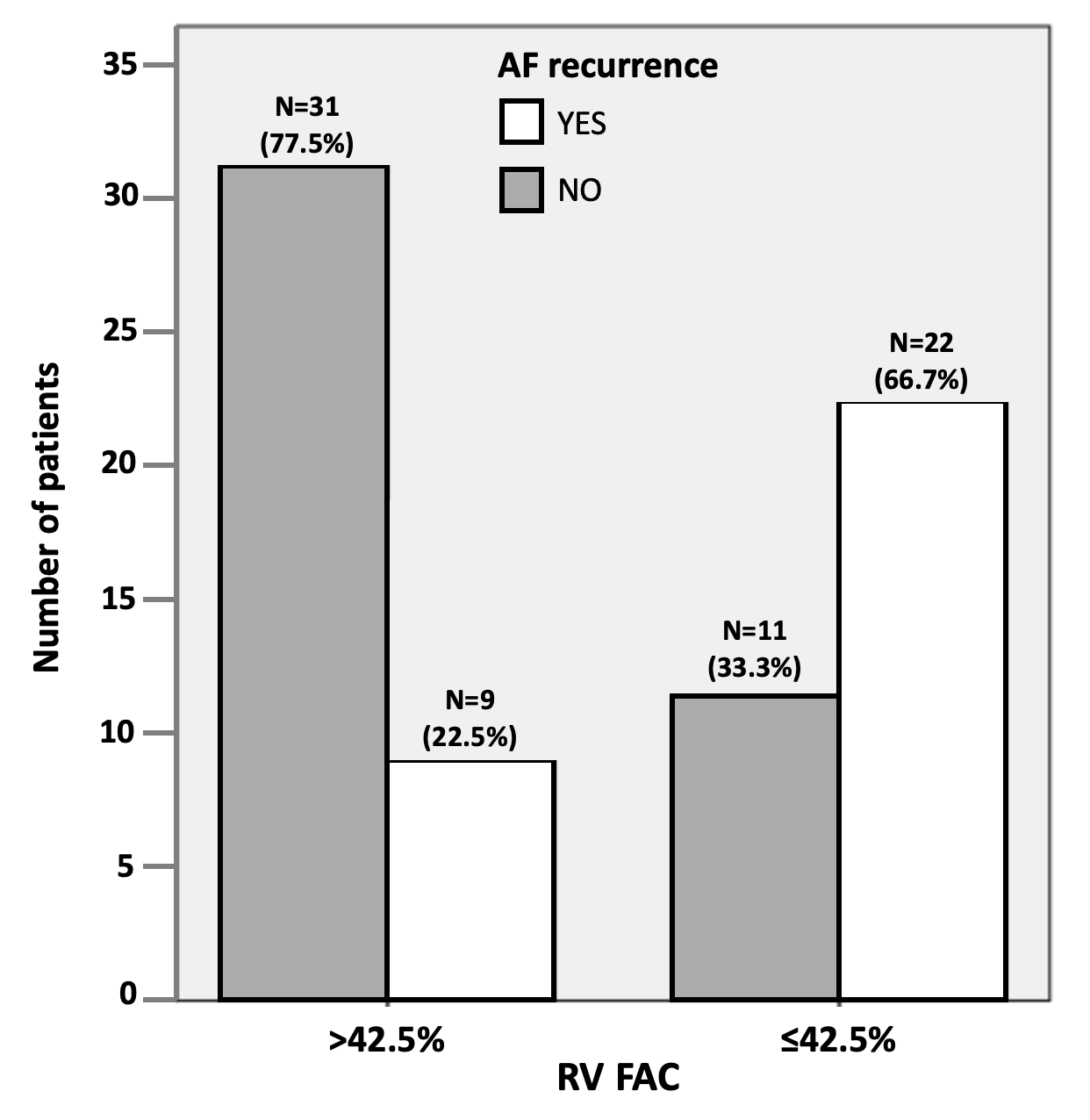

Supplement: Supplementary file 1 [file jcdd-13-00269-s001.zip › Supplementary Figure S2.png]

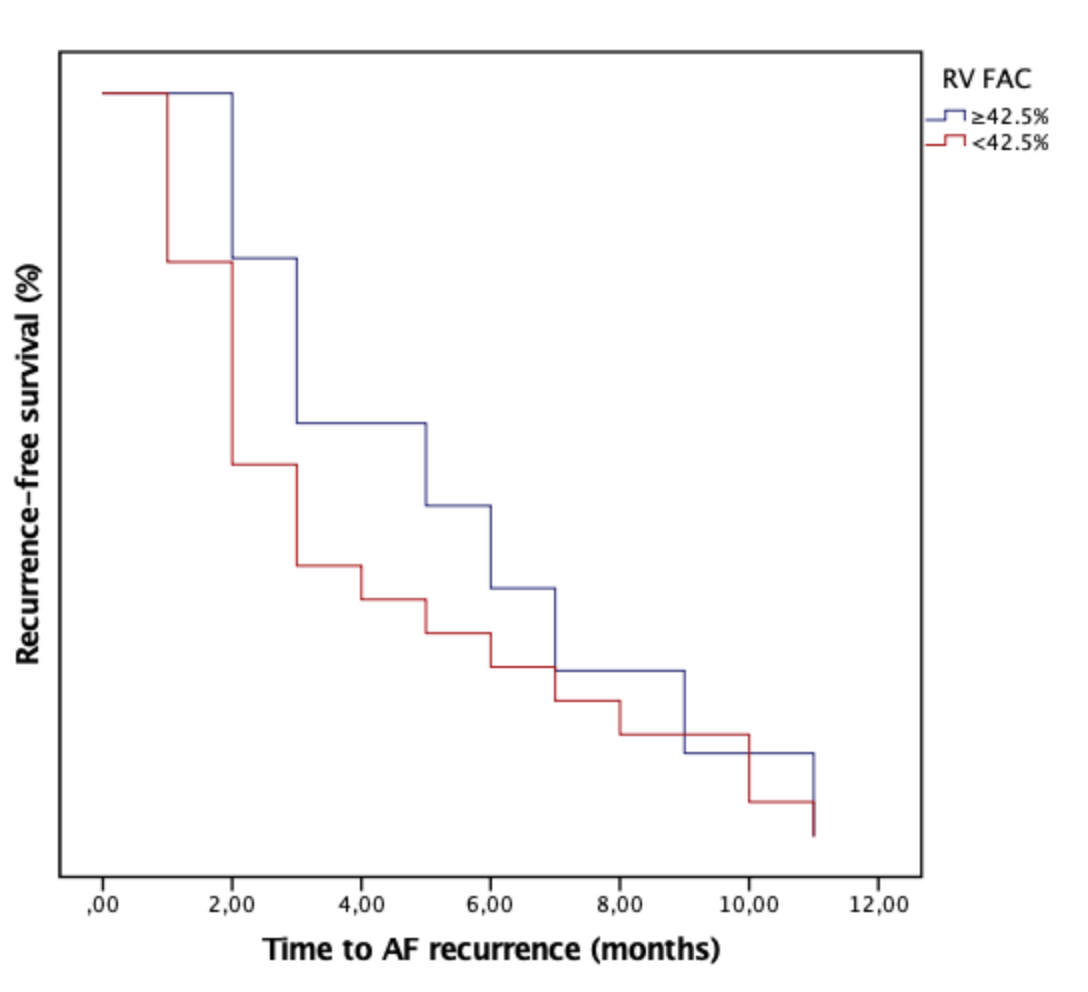

Supplement: Supplementary file 1 [file jcdd-13-00269-s001.zip › Supplementary Figure S1.png]
